# Pathobiology and management of prostate cancer-induced bone pain: recent insights and future treatments

**DOI:** 10.1007/s10787-013-0183-7

**Published:** 2013-08-06

**Authors:** Arjun Muralidharan, Maree T. Smith

**Affiliations:** 1 Centre for Integrated Preclinical Drug Development, The University of Queensland, Level 3, Steele Building, St Lucia Campus, Brisbane, QLD 4072 Australia; 2 The School of Pharmacy, The University of Queensland, Level 3, Steele Building, St Lucia Campus, Brisbane, QLD 4072 Australia

**Keywords:** Bone pain, Prostate cancer, Analgesics, Bone metastases

## Abstract

Prostate cancer (PCa) has a high propensity for metastasis to bone. Despite the availability of multiple treatment options for relief of PCa-induced bone pain (PCIBP), satisfactory relief of intractable pain in patients with advanced bony metastases is challenging for the clinicians because currently available analgesic drugs are often limited by poor efficacy and/or dose-limiting side effects. Rodent models developed in the past decade show that the pathobiology of PCIBP comprises elements of inflammatory, neuropathic and ischemic pain arising from ectopic sprouting and sensitization of sensory nerve fibres within PCa-invaded bones. In addition, at the cellular level, PCIBP is underpinned by dynamic cross talk between metastatic PCa cells, cellular components of the bone matrix, factors associated with the bone microenvironment as well as peripheral components of the somatosensory system. These insights are aligned with the clinical management of PCIBP involving use of a multimodal treatment approach comprising analgesic agents (opioids, NSAIDs), radiotherapy, radioisotopes, cancer chemotherapy agents and bisphosphonates. However, a major drawback of most rodent models of PCIBP is their short-term applicability due to ethical concerns. Thus, it has been difficult to gain insight into the mal(adaptive) neuroplastic changes occurring at multiple levels of the somatosensory system that likely contribute to intractable pain at the advanced stages of metastatic disease. Specifically, the functional responsiveness of noxious circuitry as well as the neurochemical signature of a broad array of pro-hyperalgesic mediators in the dorsal root ganglia and spinal cord of rodent models of PCIBP is relatively poorly characterized. Hence, recent work from our laboratory to develop a protocol for an optimized rat model of PCIBP will enable these knowledge gaps to be addressed as well as identification of novel targets for drug discovery programs aimed at producing new analgesics for the improved relief of intractable PCIBP.

## Introduction

Prostate cancer (PCa) is the second most common form of cancer affecting men worldwide (Ferlay et al. 2010) and a typical feature is its ability to metastasize to bone. Although metastatic cancer cells may theoretically invade any organ of the body, postmortem examination reveals that ~70 % of patients with metastatic prostate carcinomas have a high incidence of bone lesions (Coleman 2006). Metastatic bone disease in advanced-stage PCa increases the risk of intractable cancer-induced bone pain, pathological skeletal fracture, spinal-cord compression, decreased survival and poor quality of life (Coleman 2006). If PCa is detected and treated at an early stage, the 5-year survival rate is 100 % whereas if the initial diagnosis is of advanced metastatic disease, the 5-year survival rate is only 33 % (Jemal et al. 2007).

Following metastatic spread of PCa to the skeleton, patients report that persistent prostate cancer-induced bone pain (PCIBP) is one of the most distressing symptoms (Mantyh 2006). Pharmacological management of PCIBP involves use of analgesic agents such as non-steroidal anti-inflammatory drugs (NSAIDs) and opioid analgesics in combination with adjuvant therapies including bisphosphonates, corticosteroids, chemotherapy agents, radiotherapy and radionucleotides (Mercadante and Fulfaro 2007). According to the three-step ‘Analgesic Ladder’ first published by the World Health Organisation (WHO) in 1986, cancer pain should be managed according to its intensity (WHO 1986). For mild pain (Step 1), non-opioid analgesics such as NSAIDs are recommended with addition of adjuvant drugs such as anti-convulsants or tricyclic anti-depressants if there is a neuropathic component. For moderate pain (Step 2), weak opioid analgesics such as tramadol or codeine are added. For patients with moderate to severe pain (Step 3), strong opioid analgesics such as morphine are recommended together with non-opioid analgesics and adjuvant drugs if there is a neuropathic component. As NSAIDS, opioid analgesics and many adjuvants often have unacceptable side effects that may be dose limiting (IASP 2009), there is a large unmet medical need for a new generation of highly effective, well-tolerated novel analgesics/adjuvants for improved relief of PCIBP.

## Normal bone physiology

**Fig. 1:**
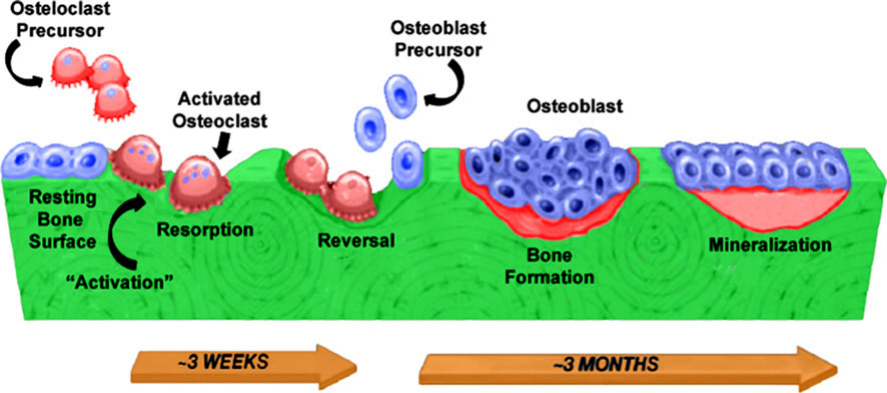
Normal bone remodelling process [adapted from Lipton (2010)]

Two vital functions of the skeleton are calcium homeostasis and mechanical support. The structural integrity of a healthy bone is maintained by a process of perpetual remodelling (Clarke 2008) encompassing removal of discrete parts of an old bone, replacement with newly synthesized proteinaceous matrix and subsequent mineralization of the matrix to form new bone (Fig. 1) (Proff and Romer 2009). The bone remodelling unit comprises a tightly coupled group of osteoclasts and osteoblasts that sequentially carry out balanced resorption and formation of bone (Saylor and Smith 2010) to prevent accumulation of bone microdamage (Proff and Romer 2009).

### Osteoclasts and osteoblasts

Osteoclasts are the only cells that are known to resorb bone. Activated multinucleated osteoclasts are derived from mononuclear precursor cells of the monocyte macrophage lineage (Takahashi et al. 2002). On the other hand, osteoblasts are bone-forming cells that arise from mesenchymal stem cells that also give rise to adipocytes and muscle cells (Aubin 1998).

Osteoblasts and stromal cells produce receptor activator of NF-κB ligand (RANKL), a member of the TNF superfamily of cytokines, that interacts with the receptor activator of nuclear factor-κB (RANK) (Lacey et al. 1998) expressed on pre-osteoclasts to induce their maturation into multinucleated osteoclasts (Dougall et al. 1999). Macrophage colony-stimulating factor (M-CSF) is required for the proliferation, survival, and differentiation of osteoclast precursors, as well as osteoclast survival and the cytoskeletal rearrangement required for bone resorption (Hattersley et al. 1991). A transcription factor that is critical for the differentiation of osteoblasts is Runt-related transcription factor 2 (Runx-2), or core-binding factor α 1 (CBFA1) (Pratap et al. 2011). Many other factors such as platelet-derived growth factor (PDGF), fibroblast growth factor (FGF), and transforming growth factor-β (TGF-β) can also enhance the growth and differentiation of osteoblasts (Mundy et al. 2001).

### Bone, a preferred site for metastases

Factors contributing to the predilection of PCa metastasis to bone include higher blood flow in the areas of red marrow, and the fact that tumour cells produce adhesion molecules such as α4β1, α5β1, αvβ3, αvβ5 that facilitate binding to marrow stromal cells and bone matrix (Lee et al. 2011). Increased production of angiogenic factors and bone-resorbing factors further enhance tumour growth in bone (van der Pluijm et al. 2001). The physical properties of the bone matrix, including low oxygen, acidic pH, high extracellular Ca^2+^ concentration (Morrissey and Vessella 2007) and growth factors such as TGF-β, insulin-like growth factors (IGF) I and II, FGF, PDGF, bone morphogenetic proteins (BMPs) (Bussard et al. 2008), which are released and activated during bone remodelling provide fertile ground for growth of the tumour cell.

## Rodent models of PCIBP

Rodent models of PCIBP involving intra-osseous injection of PCa cells with subsequent temporal development of hypersensitivity (pain) behaviours (Table 1) have been invaluable for generation of knowledge on the pathobiology of PCIBP and for the screening of novel molecules as potential analgesics/adjuvant agents for improved relief of this condition.

**Table 1:** Rodent models of prostate cancer-induced bone pain

Rodent	Gender/species	PCa cell line/concentration/route of administration	Pain/nocifensive behaviours				Additional comments	References
			Mechanical allodynia	Mechanical hyperalgesia	Thermal hyperalgesia	Guarding/flinching		
Mice	Male/athymic nude mice	10^5^ ACE-1 cells in 20 μl Hanks solution/IFI	–	NA	–	+ (Days 10–26 post-IFI)	Temporal development of osteoblastic tumours confined to the injected femur	Halvorson et al. (2005), Jimenez-Andrade et al. (2010a)
Rats	Male/copenhagen	1 × 10^6^ MAT-Ly-Lu cells in 0.1 ml PBS/IFI	NA	+ (Days 7–13 post-IFI)	NA	NA	Osteolytic damage of the distal epiphysis of the PCa-injected femur may have facilitated escape of PCa cells	De Ciantis et al. (2010)
		1 × 10^5^ MAT-Ly-Lu cells in 0.1 ml Hanks’s/IFI	NA	NA	NA	+	Local swelling (knee area) and signs of motor disablement observed in the injected hind limb	Liepe et al. (2005)
	Male/copenhagen	3 × 10^5^ AT-3.1 cells in 10 μl Hanks solution/ITI	+ (Days 13–20 post-ITI)	+ (Days 15–19 post-ITI)	NA	NA	PCa cell metastases in the adjacent tissues to the injected tibial bone resulted in temporal reduction in body weights of PCa-inject rats, c.f. sham-rats	Zhang et al. (2005)
	Male/Wistar	5 × 10^5^ AT3B-1 cells in 10 μl PBS/ITI	+ (Days 13–20 post-ITI)	–	+ (Days 13–23 post-ITI)	NA		Kolosov et al. (2011)
	Female/Dunning	1 × 10^5^ MAT-Ly-Lu cells in 0.1 ml Hanks’s/ITI	+ (Days 10–14 post-ITI)	NA	NA	NA	Significant reduction in the tibial bone mineral density between days 3 and 14 post-ITI, indicating development of osteolytic metastases	Roudier et al. (2005)

*IFI* intra-femur injection, *ITI* intra-tibial injection, + significant, c.f. to sham-controls, − non-significant, c.f. sham-controls, *NA* not assessed

However, a major limitation of many currently utilized rodent models of PCIBP (Table 1) is that they involve intra-osseous injection of very large numbers of PCa cells which results in profound bone destruction (Lamoureux et al. 2008; De Ciantis et al. 2010; Kolosov et al. 2011; Feeley et al. 2006; Liepe et al. 2005; Zhang et al. 2005). This facilitates PCa cell metastasis formation in the adjacent soft tissues as well as the lungs and liver (Kolosov et al. 2011; Liepe et al. 2005; Luo et al. 2006). The net result is the progressive deterioration of animal health, characterized by a significant temporal decrease in body weight relative to the sham-control groups (Zhang et al. 2005; De Ciantis et al. 2010), necessitating early euthanasia due to ethical concerns.

To address this issue, our laboratory has successfully established and characterized a rat model of PCIBP involving unilateral intra-tibial injection (ITI) of the optimal number of AT3B PCa cells to produce osteosclerotic tumours confined to the injected tibial bones whilst maintaining good general animal health for at least a 90-day post-ITI period (Muralidharan et al. 2013). Although there appeared to be spontaneous resolution of hindpaw hypersensitivity between days 21 and 90 post-ITI, we further showed that the underlying pain phenotype could be unmasked by administration of the prototypic opioid receptor antagonist, naloxone, at days 28–37 and 85–90 post-ITI (Muralidharan et al. 2013). Hence, our work using the aforementioned optimized rat model of PCIBP implicates a role for upregulated endogenous opioidergic signalling in masking pain associated with advanced PCa-induced bony metastases (Muralidharan et al. 2013). Importantly, our findings may explain at least in part why pain symptoms are often hidden in patients with metastatic PCa bone tumours until the later stages of metastatic disease as well as why it is often difficult for the clinicians to correlate the degree of pain reported with the functional ability of patients with disseminated PCa-induced metastases of the skeleton (Clare et al. 2005).

### Mechanisms of PCa metastasis to the bone

Skeletal metastases are radiographically classified as osteosclerotic or osteolytic (Kingsley et al. 2007). These lesions result from an imbalance between osteoblast-mediated bone formation and osteoclast-mediated bone resorption (Chirgwin and Guise 2007). The lesion is called osteosclerotic when bone formation overcomes bone resorption, or osteolytic when a decrease in bone density occurs via increased bone resorption (Kakonen and Mundy 2003). Osteolytic and osteosclerotic metastases are characteristic of breast and prostate cancer, respectively. Approximately 80 % of patients with stage IV metastatic breast cancer have osteolytic lesions (Kozlow and Guise 2005) whereas 91 % of bone metastases from prostate cancer have osteosclerotic features on plain radiography (Berruti et al. 1999). However, histology shows that the majority of PCa-induced bone metastases in patients are phenotypically heterogeneous within and between lesions although predominantly osteosclerotic (Msaouel et al. 2008).

### Metastatic process: tumour dissemination to establishment

**Fig. 2:**
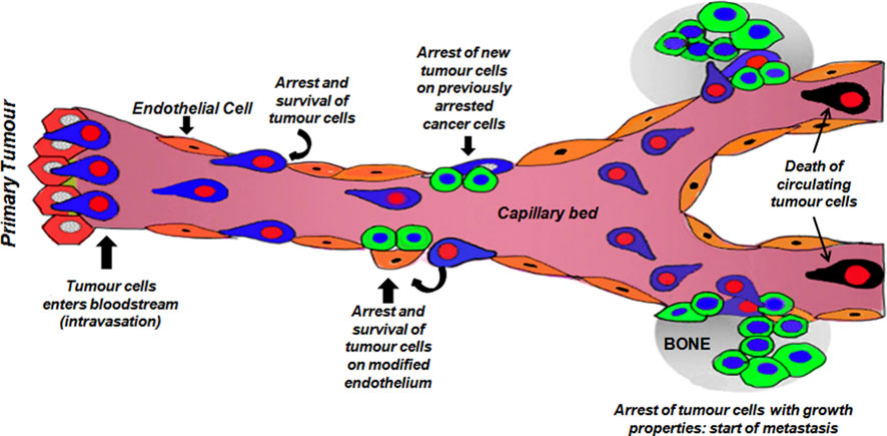
Metastatic process: tumour dissemination to establishment [adapted from Bidard et al. (2008)]

Bone metastasis is complex and begins with tropism of cells to invasion and proliferation (Fig. 2). Briefly, cancer cells detach from the primary tumour and migrate locally to invade blood vessels and the lymphatic system (intravasation). Once in the bloodstream, cancer cells are attracted to preferred sites of metastasis through site-specific interactions between tumour cells and cells in the target tissue (Weilbaecher et al. 2011).

Several groups have demonstrated the importance of the chemokine CXCL12 [also known as stromal cell-derived factor 1 (SDF-1)] and its receptor CXCR4 in PCa cell proliferation in vivo (Zhang et al. 2008b; Sun et al. 2003) and in vitro (Taichman et al. 2002; Hirbe et al. 2010). The binding of CXCL12 to CXCR4 initiates divergent downstream signalling pathways, which in turn modulate multiple aspects of tumour progression including angiogenesis, chemotaxis, cell survival and/or proliferation (Teicher and Fricker 2010). One hypothesis is that osteoblasts express the chemokine CXCL12 as well as other cytokines and growth factors including interleukin (IL)-1β, PDGF, vascular endothelial growth factor (VEGF) and tumour necrosis factor (TNF)-α that act on osteoblasts to increase CXCL12 expression levels (Jung et al. 2006). In support of this notion, at least 23 different types of cancer cells including PCa cells express CXCR4 (Balkwill 2004) and once in the bloodstream, PCa cells migrate down the chemotactic gradient to bone (Sun et al. 2005). In other work, the monocyte chemotactic protein-1 (MCP-1)/CCR2 axis has also been implicated in the tropism of PCa cells towards bone (Lu et al. 2009).

Following intravasation, cancer cells have to survive the mechanical stress of vascular transportation as well as the host’s immune defences. To survive the mechanical stress of transport in the bloodstream, cancer cells circulate as part of a fibrin clot (Walz and Fenton 1994) and to evade the immune system, there is downregulation of the expression of major histocompatibility complex (MHC) class I (Wu et al. 2004). Tumour cells that survive these hurdles adhere to the endosteal surface of bone, for which the success rate is as low as 0.1 % for each circulating cell (Luzzi et al. 1998). Factors implicated in cell adhesion and migration of PCa cells include E-selectin and sialylated glycoconjugates, vascular cell adhesion molecule-1 (VCAM-1), α_v_β_3_, α_2_β_1_, α_4_β_6_ and α_4_β_1_ integrins, cadherin-11 as well as extracellular matrix proteins (osteonectin, osteopontin, osteocalcin, bone sialoprotein and fibronectin) and the CXCL13–CXCR5 axis (Jin et al. 2011). The invading cells acquire ‘bone-cell’-like properties or ‘osteomimicry’ and produce transcription factors such as Runx2 (Pratap et al. 2011) and Homeo box homolog 2 (MSX-2) (Barnes et al. 2003) that increase the expression of osteopontin (Desai et al. 2007), osteocalcin (Huang et al. 2005), osteonectin (Campo McKnight et al. 2006) and bone sialoprotein II (Adwan et al. 2004).

#### Transition: orchestration by osteoclasts domination by osteoblasts

##### Orchestration by osteoclasts

Unlike other metastatic tumours in bone that are characterized by an ongoing vicious osteolytic cycle, PCa cells initially display osteolytic activity that eventually transforms to the predominant osteosclerotic phenotype (Msaouel et al. 2008). Hence, for patients with PCa-induced bony metastases, markers of both bone resorption [urinary N-telopeptide (uNTX), C-telopeptide type I collagen, pyridinoline cross-linked peptide and deoxypyridinoline cross-linked peptide] and bone formation [osteocalcin and bone-specific alkaline phosphatase (BAP), prostate-specific antigen (PSA), tartrate-resistant acid phosphatase (TRAP)] are high (Leeming et al. 2008; Seibel 2008; Saad and Lipton 2010a).

**Fig. 3:**
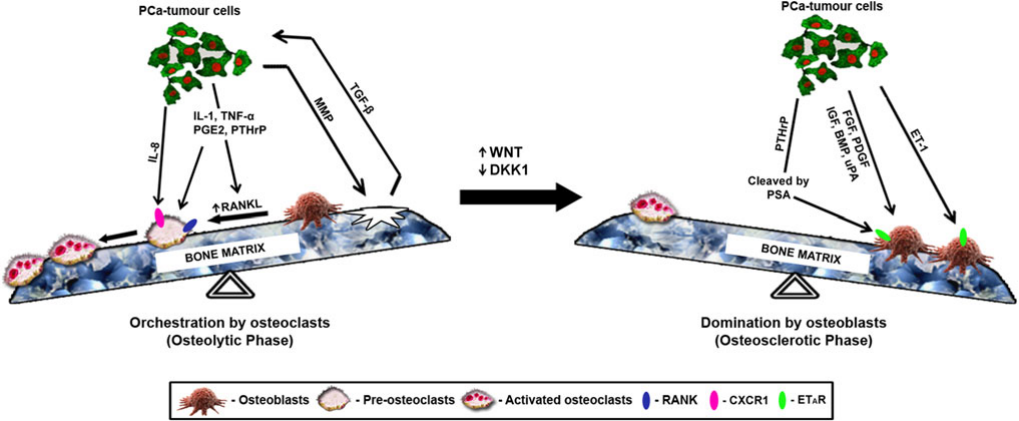
Schematic representation of the phases of phenotypic transition: orchestration by osteoclasts to domination by osteoblasts [adapted from Clines and Guise (2008)]. *Wnt* wingless-type protein, *DKK-1* dickkopf homologue 1, *PCa* prostate cancer, *TGF-β* transforming growth factor-β, *MMP* matrix metalloproteinases, *TNFα* tumour necrosis factor-α, *IL* interleukin, *PGE*_*2*_ prostaglandin E_2_, *RANKL* receptor activator of NF-κB ligand, *RANK* receptor activator of NF-κB, *PTHrP* parathyroid hormone-related protein, *FGF* fibroblast growth factor, *BMP* bone morphogenetic protein, *PDGF* platelet-derived growth factor, *IGF* insulin-like growth factor, *ET-1* endothelin-1, *uPA* urokinase-type plasminogen activator, *PSA* prostate-specific antigen

On plain radiography, PCa metastasis results in increased abnormal bone formation with an often elevated osteoid surface area and volume (Ibrahim et al. 2010). The osteolytic–osteogenic bone lesions are responsible for the abnormal bone formation and fractures at later stages (Ye et al. 2007). The initial osteolytic phase in PCa metastases helps in debulking the bone, thereby promoting seeding of cancer cells and production of growth factors (Msaouel et al. 2008). Several osteoclastogenic factors have been implicated in the increased activity of osteoclasts (Roodman 2001) including IL-1, IL-6, IL-8, IL-11, macrophage inflammatory protein 1α (MIP-1α), TNF-α, RANKL, parathyroid hormone-related protein (PTHrP) and prostaglandin E2 (PGE2) (Zhang et al. 2010; Casimiro et al. 2009; Bussard et al. 2008) (Fig. 3). Of interest, many of these factors are pro-inflammatory and are implicated in the pathobiology of multiple chronic pain states (see “Metastasis or tumourigenesis inhibitors” for further discussion).

Osteoprotegerin (OPG), a natural decoy receptor for RANKL, acts as a negative regulator of the RANK/RANKL pathway by sequestering RANKL (Lipton and Goessl 2011). Though several other hormones and cytokines may play a role, only RANKL is absolutely required for osteoclastogenesis (Li et al. 2000). PCa cells express RANK/RANKL and the levels of RANKL/OPG are elevated in patients with PCa-induced bone metastases (Chen et al. 2006). OPG also serves as a survival factor by inhibiting the apoptosis of PCa cells induced by the TNF-related apoptosis-inducing ligand (TRAIL) (Holen et al. 2002).

Apart from direct stimulation of osteoclast precursors, IL-1, TNF-α, PGE2 and PTHrP contribute to osteoclastogenesis by upregulating the production of RANKL by osteoblasts (Kwan Tat et al. 2004). In addition, TGF-β released as a result of bone matrix degradation by matrix metalloproteinases (MMPs) stimulates PTHrP, thereby creating a positive feedback loop (Guise and Chirgwin 2003). IL-8 stimulates osteoclastogenesis via increasing RANKL production by binding to the chemokine receptor, CXCR1, on osteoclast precursor cells (Bendre et al. 2003). This in turn increases PGE2 (Kundu et al. 2001) and suppresses osteoblast activity (Dovio et al. 2004). MIP-1α is a potent inducer of osteoclast formation in vitro in a manner independent of RANKL that enhances both RANKL-stimulated and IL-6-stimulated osteoclast formation (Han et al. 2001).

##### Domination by osteoblasts

Metastasis factors such as wingless-type protein (Wnt)-1, IGF-I, BMPs, basic fibroblast growth factor (bFGF), IL-6, endothelin (ET)-1 and PTHrP dominate by stimulating osteoblast activity through autocrine and paracrine activity (Ibrahim et al. 2010). The Wnt pathway/ET axis/BMP pathway has emerged as a key regulator of osteosclerotic metastasis (Robinson et al. 2008; Sethi and Kang 2011) (Fig. 3).

Wnt proteins are soluble glycoproteins that bind to frizzled G protein-coupled receptors and low-density lipoprotein receptor-related protein (Bodine and Komm 2006). Wnt signalling is a key osteoblast regulatory pathway critical for osteoblast differentiation and function (Bodine and Komm 2006; Sethi and Kang 2011). Dickkopf homologue 1 (DKK-1) is a protein that regulates PCa bone metastasis progression by opposing the actions of osteogenic Wnts early on, thereby controlling the phenotypic switch from osteolytic to osteosclerotic lesions (Hall et al. 2008). Indeed, levels of DKK-1 are elevated in early events associated with PCa but levels decrease with disease progression, thereby increasing osteosclerotic activity in advanced metastases (Hall et al. 2008).

ET-1 is implicated as a central mediator of osteosclerotic metastasis (Mohammad and Guise 2003) as it stimulates the formation of bone and the proliferation of osteoblasts that express the endothelin A receptor (ET_A_R) (Kasperk et al. 1997). Levels of alkaline phosphatase, a bone formation marker, are elevated in patients with osteosclerotic PCa cell-induced bone metastases (Nelson et al. 1995). Although PTHrP is an osteolytic factor, it is highly expressed even in the later stages of PCa. An explanation for this somewhat paradoxical observation is that the NH_2_-terminal fragments of PTHrP that are cleaved by PSA (Cramer et al. 1996) share high sequence homology with ET-1 and therefore likely activate the ET_A_R (Schluter et al. 2001). ET-1 is thought to activate the Wnt signalling pathway by reducing paracrine production of the Wnt antagonist, DKK-1 (Clines et al. 2007).

Other factors including PDGF (Yi et al. 2002), urokinase-type plasminogen activator (uPA) (Achbarou et al. 1994), PSA (Cramer et al. 1996), IGF-binding proteins (IGFBP) and BMP-2/6/7 also play a significant role (Casimiro et al. 2009). The overproduction of uPA by prostate cancer cells increases the severity of bone metastasis (Achbarou et al. 1994). Prostate cancer cells also release PSA, a kallikrein serine protease. In addition to cleaving PTHrP, PSA also cleaves IGFBP-3, thereby paving the way for IGF-1 to bind to its receptor and to stimulate osteoblast proliferation (Cohen et al. 1994). Thus, there are many tumour-produced factors that impact osteoclasts, osteoblasts, the tumour itself and the bone microenvironment in a vicious cycle to promote the development and progression of bone metastasis (Buijs et al. 2007).

## Pathophysiology of PCIBP

### Nociceptive signalling and pain

Pain severity reports by patients comprise an integration of nociception overlaid by emotional interpretation by higher centres in the brain (Rainville et al. 1997). Briefly, nociception involves detection of potentially damaging stimuli by free nerve endings (nociceptors) in the periphery to generate action potentials that are transmitted by primary afferent sensory nerve fibres to laminae I and II of the dorsal horn of the spinal cord (Sherrington 1906). Transmission of these nociceptive signals by second-order neurons via the spinothalamic tracts to higher centres in the brain may in turn activate endogenous descending opioidergic and noradrenergic signalling pathways to reduce pain severity (see reviews by Smith and Muralidharan 2013; Basbaum et al. 2009). In chronic inflammatory and peripheral neuropathic pain states, peripheral nociceptors become sensitized resulting in ectopic firing and induction of neuroplastic changes in the spinal cord and supraspinally; this has been reviewed in detail elsewhere (see Smith and Muralidharan 2013; Basbaum et al. 2009).

In the following sections of our review herein, we discuss the mechanisms underpinning sensitization of peripheral nerve fibres and the development of so-called ‘central sensitization’ in the context of the development and maintenance of PCIBP.

### Peripheral sensitization mechanisms in PCIBP

In patients with advanced bony metastases, intermittent episodes of extreme pain, known as breakthrough pain, may occur spontaneously as a result of bone remodelling (Mercadante 1997) or be induced by movement of tumour-bearing bone (Mercadante and Arcuri 1998). The various compartments of the bone, viz. bone marrow, mineralized bone and the periosteum, are densely innervated by both sensory and/or sympathetic nerve fibres (Jimenez-Andrade et al. 2010b). Thus, tumours invading and proliferating within the medullary space of the bone sensitize primary afferent nerve fibres and induce pronounced infiltration of inflammatory cells. The periosteum is innervated by a mesh of calcitonin gene-related peptide (CGRP) and substance P (Sub P)-expressing sensory nerve fibres that are implicated in movement-related pain (Martin et al. 2007).

**Fig. 4:**
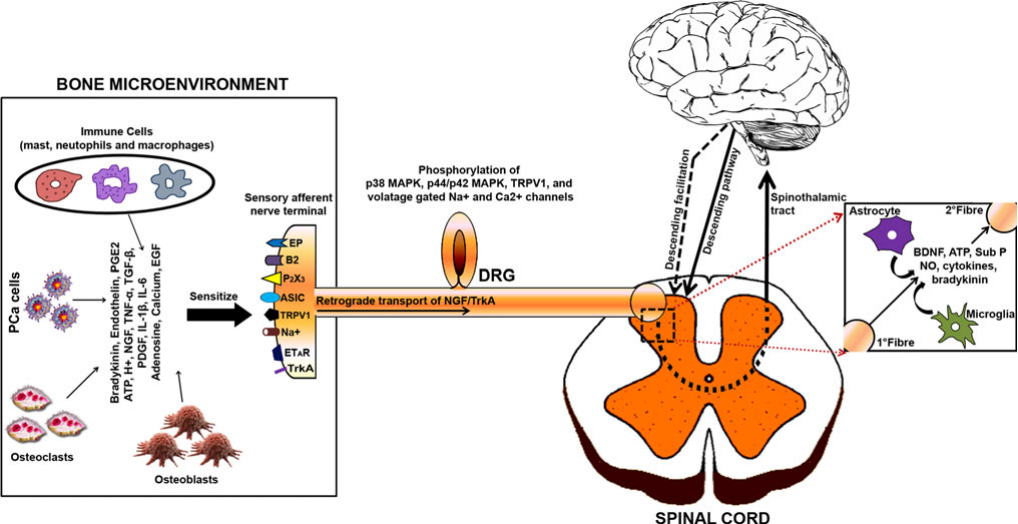
Pathophysiology of cancer-induced bone pain [adapted from Smith and Muralidharan (2013)]. *IL* Interleukin, *NGF* nerve growth factor, *TNF* tumour necrosis factor, *ATP* adenosine triphosphate, *H* ^+^ hydrogen ion, *PGE2* prostaglandin, *TGF-β* transforming growth factor, *PDGF* platelet-derived growth factor, *EGF* epidermal growth factor, *Na* ^+^ sodium ion channel, *B2* bradykinin receptor, *P2X3* purinergic receptor, *ASIC* acid-sensing ion channel, *EP* prostaglandin receptor, *ET*_*A*_ *R* endothelin A receptor, *TrkA* tyrosine kinase A, *TRPV1* transient receptor potential vanilloid 1, *SubP* substance P, *BDNF* brain-derived neurotropic factor, *NO* nitric oxide

The broad array of pro-hyperalgesic mediators released by osteoclasts, osteoblasts, tumour and tumour-associated immune cells (macrophages, neutrophils and T cells) outlined in the preceding section (“Transition: orchestration by osteoclasts domination by osteoblasts”) collectively sensitize peripheral nociceptors in the bone milieu to subsequent nociceptive stimuli and/or directly activate specific receptors located on the primary afferents themselves (Jimenez-Andrade et al. 2010b). Thus, prostaglandins, endothelins, bradykinin, colony-stimulating factors, TNF-α, TGF-β, PDGF, IL-1, nerve growth factor (NGF) and IL-6 are components of a ‘pro-inflammatory soup’ that sensitize nociceptors in prostate cancer-induced bone pain (Fig. 4) (Mantyh 2006; Schmidt et al. 2010). In addition, osteoclast- and tumour-induced acidosis in bone tissue may also contribute to the pathobiology of PCIBP by sensitizing subsets of sensory neurons that express the transient receptor potential vanilloid 1 (TRPV1) (Caterina et al. 2000) and/or the acid-sensing ion channel 3 (ASIC3) (Olson et al. 1998).

Following injection of PCa cells into the mouse femur, profound pathological sprouting of CGRP^+^ and neurofilament 200 kDa (NF200)^+^ sensory nerve fibres and tyrosine hydroxylase-positive post-ganglionic sympathetic nerve fibres are induced (Jimenez-Andrade et al. 2010a) in close proximity to colonies of PCa cells, tumour-associated stromal cells and newly formed woven bone, which together form osteosclerotic lesions (Jimenez-Andrade et al. 2010a). This ectopic sensory nerve fibre sprouting appears to be induced particularly by the tumour-associated stromal cells and confined to sensory fibres that co-express the high-affinity NGF receptor, tyrosine kinase (Trk) A receptor (TrkA) (Jimenez-Andrade et al. 2010a). Retrograde transport of NGF via the peripheral terminals of abnormally sprouting sensory nerve fibres to the cell bodies of primary sensory neurons in the dorsal root ganglia (DRGs) likely contributes to first-order sensory neuron hyperexcitability via multiple mechanisms. Such mechanisms include upregulated synthesis of pro-nociceptive mediators (Mantyh et al. 2011), activation of p38 mitogen-activated protein kinase (MAPK) (Ji et al. 2002) and p44/p42 MAPK (Averill et al. 2001)-induced sensitization (phosphorylation) of the TRPV1 (Ji et al. 2002) as well as voltage-gated sodium (Hudmon et al. 2008; Stamboulian et al. 2010) and calcium channels (Martin et al. 2006). Together, these observations strongly implicate a role for NGF/TrkA signalling in the maintenance of PCIBP. In further support of this notion, preventive or late administration of an anti-NGF antibody to mice with PCa cell-induced tumours in the femur significantly attenuated PCIBP by blocking tumour-induced ectopic nerve fibre sprouting and neuroma formation in the bone periosteum (Jimenez-Andrade et al. 2011; Halvorson et al. 2005).

Interestingly, NGF-dependent nerve fibre changes observed in PCIBP (Jimenez-Andrade et al. 2011; Halvorson et al. 2005) have also been shown in rodent models of peripheral nerve injury-induced neuropathic pain (Peleshok and Ribeiro-da-Silva 2011; Zhang and Strong 2008). For example, in rats with sciatic nerve injury, axonal sprouting of injured sciatic nerve sensory fibres was correlated with peak hindpaw hypersensitivity (Sommer et al. 1995) as well as with sympathetic nerve fibre sprouting in glabrous skin (Yen et al. 2006). In other work, unilateral sciatic nerve administration of NGF in non-injured rats induced dose-dependent thermal hyperalgesia together with demyelination and axonal sprouting to mimic the changes seen in nerve-injured rats (Ruiz et al. 2004). Following sciatic nerve transection (SNT) in rats, local administration of an NGF-sequestering fusion protein (TrkA-IgG) at the site of nerve transection blocked neuroma formation as well as the development of neuropathic pain behaviours (Kryger et al. 2001). Importantly, as there were no significant changes in the cellular characteristics of the DRGs of SNT-rats that received TrkA-IgG treatment, c.f. control rats, TrkA-IgG appears to act only at the local site of nerve transection (Kryger et al. 2001).

### Central pain mechanisms

Tissue inflammation or peripheral nerve injury-induced sensitization of peripheral sensory nerve fibres results in their hyperexcitability, characterized by ectopic discharge to induce so-called ‘central sensitization’ in the dorsal horn of the spinal cord and supraspinally. Central sensitization is underpinned by multiple neuroplastic changes in the functional responsiveness of nociceptive circuits due to increased membrane excitability and/or reduced inhibition in the spinal cord (Gordon-Williams and Dickenson 2007). In rodent models of inflammatory and neuropathic pain, augmented glutamate signalling via *N*-Methyl-d-aspartic acid (NMDA), α-amino-3-hydroxy-5-methyl-4-isoxazolepropionic acid receptor (AMPAR), group I–III metabotropic glutamate receptors (mGluR), as well as brain-derived neurotrophic factor (BDNF), Sub P, CGRP, nitric oxide (NO) and bradykinin have all been implicated in the mechanisms underpinning central sensitization (Latremoliere and Woolf 2009). In addition, degeneration of inhibitory GABAergic interneurons in the spinal cord (Scholz et al. 2005) and/or enhanced descending 5-HT_3_-mediated facilitation (Gordon-Williams and Dickenson 2007) may contribute to central sensitization. Apart from neuronal changes, peripheral nerve injury also induces activation of microglia and astrocytes in the CNS, which under normal conditions perform “house-keeper” roles to support on-going function and survival of neurons. Once activated, microglia and astrocytes release multiple pronociceptive substances including cytokines, chemokines, neurotrophic factors, adenosine triphosphate (ATP), NO and excitatory amino acids that enhance pain by amplifying CNS neuron hyperexcitability (Fig. 4) (Vallejo et al. 2010).

In a rat model of PCIBP, activation of microglia and astrocytes together with upregulation of IL-1β developed in the ipsilateral spinal cord of rats exhibiting pain behaviours at 20 days after unilateral ITI of rat prostate cancer cells (Zhang et al. 2005). In other work, IL-1β facilitated PCIBP by enhancing phosphorylation of the NR1 subunit of the NMDA receptor whereas pain behaviour was blocked by treatment with an IL-1 receptor antagonist (Zhang et al. 2008a).

### Knowledge gaps in the pathobiology of PCIBP

The recent advent of rodent models of PCIBP has enabled the relative contributions of invading tumour cells vis-a-vis factors related to the bone microenvironment, to the pathogenesis of PCIBP to be examined. However, additional research is needed to elucidate the biochemical and molecular mechanisms that underlie cross talk between these various aspects of PCIBP.

Research using murine models of breast cancer- and osteolytic sarcoma-induced bone pain implicates a unique functional responsiveness of the noxious circuitry in spinal cord sensitization (Table 2). This is characterized by a neurochemical signature of pro-hyperalgesic mediators in the DRGs (Table 3) and the dorsal horn of the spinal cord (Table 4) that differ from those for neuropathic and/or inflammatory pain. For example, in fibrosarcoma-bearing mice, the proportion of wide dynamic range (WDR) neurons was unchanged (Khasabov et al. 2007), whereas in breast cancer-induced bone tumour-bearing rats, the proportion of WDR neurons increased significantly (Urch et al. 2003). Hence, it is important to be aware of the potential for between-tumour differences in the mechanisms underpinning central sensitization in the spinal cord as a result of cancer-induced bone metastases.

**Table 2:** Comparative electrophysiological findings between rodent models of peripheral nerve injury and cancer-induced bone pain

Neuropathic pain (NP) models	Breast cancer-induced bone pain	Ostelolytic fibrosarcoma-induced bone pain
Significant increase in the peripheral receptive field size in both superficial and deeper neurons of the spinal cord (Suzuki et al. 2000)	Significant increase in the peripheral receptive field size in only superficial neurons (Urch et al. 2003; Donovan-Rodriguez et al. 2004)	Significant increase in the peripheral receptive field size in only superficial neurons (Yanagisawa et al. 2010)
Increased levels of ongoing activity of both WDR and HT neurons (Sotgiu et al. 1994)	Increased levels of ongoing activity of WDR, but not HT neurons (Urch et al. 2003)	Increased levels of ongoing activity of WDR, but not HT neurons (Khasabov et al. 2007; Yanagisawa et al. 2010; Simone et al. 2008)
Increase in the proportion of WDR neurons in the NP rats (32 %), c.f. control rats (22 %) (Liu et al. 2011)	Increase in the proportion of WDR neurons in the tumour-bearing rats (47 %), c.f. control rats (26 %) (Urch et al. 2003; Donovan-Rodriguez et al. 2004)	No change in the proportion WDR neurons in tumour-bearing mice (64 %), c.f. control mice (56 %) (Khasabov et al. 2007)
Sensitization of both WDR and HT neurons contribute to mechanical stimuli (Sotgiu et al. 1995)	Sensitization of WDR, but not HT, neurons contribute to tumour-evoked mechanical stimuli (Urch et al. 2003; Donovan-Rodriguez et al. 2004)	Sensitization of WDR, but not HT, neurons contribute to tumour-evoked mechanical stimuli (Khasabov et al. 2007; Yanagisawa et al. 2010; Simone et al. 2008)
WDR neurons do not exhibit sensitization to heat stimuli (Laird and Bennett 1993)	Sensitization of WDR neurons to heat stimuli (Urch et al. 2003; Donovan-Rodriguez et al. 2004)	Sensitization of WDR neurons to heat stimuli (Simone et al. 2008; Khasabov et al. 2007)
The amplitude and frequency of sEPSCs of SG neurons were unaffected (Okamoto et al. 2001; Kohno et al. 2003)	NA	The amplitude of sEPSCs of SG neurons are increased, but their frequencies remained unchanged (Yanagisawa et al. 2010)
Increase in Aβ fiber-mediated EPSCs of SG neurons when compared with control animals (Okamoto et al. 2001; Kohno et al. 2003)	NA	No change in Aβ fiber-mediated EPSCs of SG neurons when compared with control animals (Yanagisawa et al. 2010)
Spinal sensitization is present at lumbar levels where central terminals of primary afferent sensory neurons innervate (Okamoto et al. 2001; Kohno et al. 2003)	NA	Spinal sensitization is present throughout multiple lumbar spinal levels rather than just the segments in which the central terminals of primary afferent sensory neurons innervate (Yanagisawa et al. 2010)

*sEPSCs* spontaneous excitatory postsynaptic currents, *SG* substantia gelatinosa, *WDR* wide dynamic range, *HT* high threshold neurons, *NA* not assessed

**Table 3:** Comparison of neurochemical alterations in primary afferent sensory neurons in rodent models of inflammatory pain [Freund’s complete adjuvant (CFA)], spinal nerve ligation (SNL), sciatic nerve transection (SNT) and CIBP (adapted from Honore et al. 2000b; Peters et al. 2005; Braz and Basbaum 2010; Guo et al. 2007; Kim et al. 2009a)

Makers	CFA	SNL	SNT	CIBP
Sub P	⟷	↘	↘	⟷
IB4	⟷	↘	↘	⟷
CGRP	⟷	↘	↘	⟷
GAL	⟷	↗	↗	↗
NPY	⟷	↗	↗	⟷
ATF3	⟷	↗	↗	↗
GFAP	↗	↗	↗	↗

The above symbols represent a significant increase (↗), decrease (↘) or no significant changes (↔) in the immunofluorescence of neurochemical markers in lumbar DRGs

*SP* substance P, *IB4* isolectin B4, *CGRP* calcitonin gene-related peptide, *GAL* galanin, *NPY* neuropeptide Y, *ATF3* activating transcription factor 3, *GFAP* glial fibrillary acidic protein

**Table 4:** Comparison of the neurochemistry of inflammatory pain, spinal nerve ligation- induced pain, sciatic nerve transection and bone cancer pain in the dorsal horn of the spinal cord of rodent models (adapted from Honore et al. 2000a, 2009; Schwei et al. 1999; Raghavendra et al. 2004; Lin et al. 2007)

Makers in laminae I–II	CFA	SNL	SNT	CIBP
Sub P	↗	↘	↘	⟷
IB4	⟷	↘	↘	⟷
CGRP	↗	↘	↘	⟷
GAL	⟷	↗	↗	⟷
NPY	⟷	↗	↗	⟷
DYN	⟷	⟷	↗	⟷
GFAP	↗	↗	↗	↗
OX-42	⟷	↗	⟷	⟷

The above symbols represent a significant increase (↗), decrease (↘) or no significant changes (↔) in the immunofluorescence of neurochemical markers in laminae I–II of the spinal cord

*SP* substance P, *IB4* isolectin B4, *CGRP* calcitonin gene-related peptide, *GAL* galanin, *NPY* neuropeptide Y, *ATF3* activating transcription factor 3, *GFAP* glial fibrillary acidic protein, *OX-42* microglial marker

At present, the functional responsiveness of noxious circuitry and knowledge on the neurochemical signature of pro-hyperalgesic mediators in the DRGs and spinal cord of rodent models of PCIBP is poorly understood, and so this knowledge gap needs to be addressed. An improved understanding of the neurobiology of PCIBP has the potential to identify new targets for use in drug discovery programs aimed at producing a new generation of analgesics and/or adjuvant drugs for the improved relief of intractable pain due to advanced prostate cancer-induced bone pain.

## Therapeutic strategies for the management of PCIBP

**Fig. 5:**
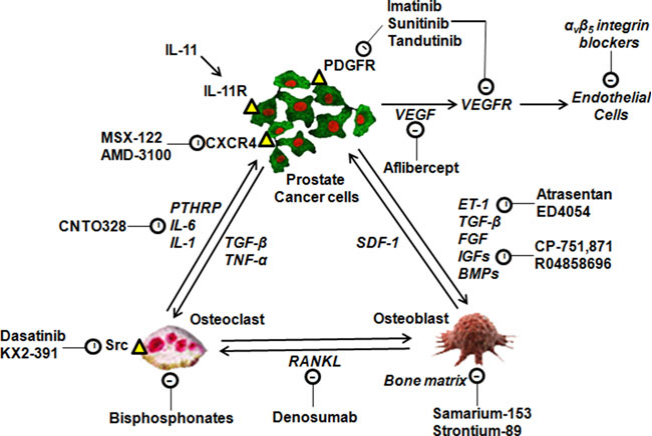
Schematic diagram summarizing a range of potential therapeutic targets for novel drugs aimed at reducing prostate cancer-induced bone metastasis [adapted from Tu and Lin (2008)]

The pathobiology of prostate cancer-induced bone pain is underpinned by neuroplastic changes at multiple levels of the somatosensory system, in addition to contributions from immune, stromal and tumour-associated factors in the bone microenvironment. Hence, it is possible that the quality of life and survival of patients with metastasis-induced PCIBP may be improved not only by analgesic agents (see reviews by Cleary 2007 and Nersesyan and Slavin 2007), but also by treatments that inhibit the tumourigenic process including metastasis to bone. Hence, an overview of novel therapeutic agents aimed at blocking progression of prostate cancer-induced metastasis to bone, that have at least reached Phase III clinical trials, is provided in the next section. A schematic diagram summarizing a range of potential therapeutic targets for novel agents aimed at reducing prostate cancer-induced bone metastasis is shown in Fig. 5.

### Therapeutic agents with defined analgesic potential

#### Radiotherapy

Approximately 20 years ago, the Radiation Therapy Oncology Group reported that 80–90 % of patients receiving radiotherapy (RT) for osseous metastases experience partial to complete pain relief within 10–14 days of RT initiation (Tong et al. 1982). Three types of RT are used for treatment of bone metastases, viz external beam radiotherapy (EBRT), hemi-body irradiation (HBI) and radiopharmaceuticals (Pandit-Taskar et al. 2004). Systematic review shows that EBRT, whether given as single or multiple fractions, produces 50 % pain relief in 41 % of patients and complete pain relief at 1 month in 24 % of patients (McQuay et al. 2000). Although HBI provides rapid pain relief, it comes at the expense of toxicity (Lin and Ray 2006). Systemic review and meta-analysis of randomized controlled clinical trials found that single-fraction radiotherapy with 1 × 8 Gy is as effective for pain relief as multi-fraction regimens such as 5 × 4 Gy in 1 week or 10 × 3 Gy in 2 weeks (Wu et al. 2003; Sze et al. 2003, 2004). More recently, a prospective study involving 91 patients with painful bone metastases who were treated with a median total dose of 46 Gy (Nomiya et al. 2010), found that complete and partial pain relief (≥50 %) were obtained in 49 and 91 % of patients, respectively (Nomiya et al. 2010). Although the optimal dose fractionation for radiation of metastatic bone lesions has been controversial, an internet survey of radiation oncologists, with members participating from the American Society for Radiology Oncology, Canadian Association of Radiation Oncology, and Royal Australian and New Zealand College of Radiologists, concluded that the most accepted fractionation schemes are 8 Gy in a single fraction and 30 Gy in ten fractions (Fairchild et al. 2009).

Radioactive isotopes of phosphorus (P)-32 and strontium (Sr)-89 were the first bone-seeking radiopharmaceuticals approved by the United States (US) Food and Drug Administration (FDA) for the treatment of painful bone metastases, followed by samarium (Sm)-153, rhenium (Re)-186, and Re-188 (Lewington 1996; Lambert and de Klerk 2006). P-32 is no longer used because of the associated myelosuppression (Lewington 1996). Sr-89 chloride (Metastron™) and Sm-153–lexidronam (Quadramet^®^) are effective for treating PCa cell-induced bone metastases (Liepe and Kotzerke 2007; Dolezal et al. 2007), with 80 % of patients with prostate cancer-induced painful osteoblastic bony metastases achieving pain relief following strontium-89 administration (Robinson et al. 1993). Concomitant administration of radiopharmaceuticals with bisphosphonates (Lam et al. 2008) and chemotherapy (Amato et al. 2008; Akerley et al. 2002; Pagliaro et al. 2003) improved patient survival and quality of life. A Cochrane review of the efficacy and safety of radioisotopes in patients with metastatic bone pain concluded that there was evidence to support their use as analgesics with a number needed to treat (NNT) to achieve complete and complete/partial pain relief at 5 and 4, respectively (Roque et al. 2011). More recently, Phase II clinical studies of the α-emitting radioisotope, radium (Ra-223), demonstrated significant improvements in overall survival, time to first skeletal-related events (SRE), pain response and biochemical parameters with very good tolerability, in men with castration-resistant prostate cancer (CRPC) metastasized to bone (Parker et al. 2013; Nilsson et al. 2012). A Phase III randomized clinical trial (ALSYMPCA) investigating the analgesic efficacy, overall survival benefit and safety profile of Ra-223 (50 kBq/kg i.v.) is currently ongoing (NCT00699751). At interim analysis, Ra-223 demonstrated significant improvements in overall survival, pain response and time to initial EBRT or opioid use (Parker et al. 2012; Nilsson et al. 2013).

#### Bisphosphonates

Bisphosphonates (BPs) are pyrophosphate analogues that bind avidly to hydroxyapatite bone mineral surfaces and are selectively internalized by osteoclasts (Russell et al. 1999), thereby disturbing the cytoskeleton and loss of actin rings leading to osteoclast apoptosis (Russell et al. 1999). The anti-proliferative, anti-angiogenic and apoptotic properties (see Clezardin 2011 for review) of BPs is supported by efficacy data from rodent models (Hall and Stoica 1994; Yoneda et al. 1997; Clohisy et al. 2001).

BPs are effective in reducing PCIBP and the occurrence of SREs, either when used alone or concomitantly with radiation therapy (Manas et al. 2008; Lilleby 2007; Yuen et al. 2006; Rodrigues et al. 2004). A Cochrane review of BPs in metastatic bone disease concluded that the NNT for analgesic efficacy was 11 at 4 weeks but reduced to 7 at 12 weeks (Wong and Wiffen 2002). Zoledronic acid (Zometa^®^, Novartis) and pamidronate (Aredia^®^, Novartis) were approved by the US FDA for the treatment of metastatic bone complications in 1995 and 2002, respectively (Saylor and Smith 2010). Although BPs may alleviate PCIBP, more research is needed to guide the choice of BPs as well as to optimize the treatment schedule (Yuen et al. 2006).

#### Non-steroidal anti-inflammatory drugs (NSAIDs)

A meta-analysis of 25 randomized controlled trials related to the use of NSAIDs in cancer pain in humans found that although NSAIDs significantly reduced cancer-related pain above placebo, it was not possible to draw conclusions on their efficacy for relief of cancer-induced bone pain as pain due to bony metastases were not reported on separately from other cancer pain (Eisenberg et al. 1994). More recently, a Cochrane review of the use of NSAIDs in 42 randomized clinical trials, either alone or in combination with opioids for the relief of cancer pain, concluded that NSAIDs were more effective than placebo, but that evidence to support the superior safety or efficacy of one NSAID over another, was lacking (McNicol et al. 2005). Despite the aforementioned limitations, NSAIDs administered either alone or in combination with opioids, are recommended for the relief of metastatic bone pain (IASP 2009). Selective COX-2 inhibitors may have therapeutic benefit due to their anti-inflammatory and anti-tumourigenic properties (Sumitani et al. 2001). In support of this notion, acute administration of selective COX-2 inhibitors to rodents with cancer-induced bone pain attenuated hypersensitivity (pain) behaviours, whereas chronic treatment reduced tumour burden, osteoclast destruction in addition to producing significant pain relief (Sabino et al. 2002).

The findings of Phase III clinical trials on the analgesic efficacy of radiopharmaceuticals, including strontium-89 (Sr-89), samarium-153 (Sm-153), rhenium-186 (Re-186) and radium-223 (Ra-223), as well as BPs such as zoledronic acid and pamidronate, in patients with prostate cancer-induced bone pain are summarized in Table 5. A detailed review of randomized clinical studies on the analgesic efficacy of radiotherapy may be found elsewhere (see Rades et al. 2010).

**Table 5:** Summary of Phase III clinical trials that assessed the analgesic efficacy of radiopharmaceuticals and bisphosphonates in patients with prostate cancer-induced bone pain

Patient and treatment description (# patients)	Primary endpoint	Results	Adverse events	References
Radiopharmaceuticals				
Patients with endocrine refractory metastatic prostate cancer	Pain response using RTOG criteria, analgesic use, QoL using Visual Analogue Scale	At 3 months, complete pain relief 50 % (Sr-89) vs. 36 % (placebo); discontinuation of analgesics 17.1 % (Sr-89) vs. 2.4 % (placebo). Addition of Sr-89 to EBRT reduced analgesic requirements	Leukopenia grade-3/4: 12 % in Sr-89 vs. 0 % in placebo; thrombocytopenia grade-3/4: 32.8 % in Sr-89 vs. 3.4 % in placebo	Porter et al. (1993)
Local EBRT plus single injection of 10.8 mCi Sr-89 (68) or placebo (58)				
Patients with painful skeletal metastases	Progression of disease (using QLQ C-30 v2.0 questionnaire, pain score, analgesic requirement, WHO performance status)	At 3 and 6 months, no differences in the disease progression between the two groups. Role of strontium-89 as adjuvant to palliative EBRT is questionable	Leukopenia grade-1/2: 36.4 % in Sr-89 vs. 13.3 % in placebo; thrombocytopenia grade-1/2: 15.9 % in Sr-89 vs. 4.4 % in placebo	Smeland et al. (2003)
10 fractions of 3 Gy EBRT plus single intravenous 150 MBq Sr-89 (46) or placebo (49)				
Patients with metastatic HRPC	Pain response and duration of response	Pain response in 91 % (Sr-89/cisplatin) vs. 63 % (Sr-89/placebo), duration of pain relief 120 days (Sr-89/cisplatin) vs. 60 days (Sr-89/placebo). Addition of a low dose of cisplatin enhances the effect of a standard dose of Sr-89	Anaemia grade-3/4: 8.5 % in Sr-89 vs. 11.4 %; leukopenia	Sciuto et al. (2002)
148 MBq Sr-89 plus 50 mg/m^2^ cisplatin (35) vs. Sr-89 plus placebo (35)			Grade-1/2: 22.9 % in Sr-89 vs. 5.7 % in placebo; thrombocytopenia grade 1/2: 2.8 % in Sr-89 vs. 5.7 % in placebo	
Patients with metastatic CRPC	Pain response, mobility and analgesic use	At 3 months, 65–70 % of patients had pain relief with Sr-89 compared to 66.7 % with local EBRT and 67.4 % with HBI. However, patients treated with Sr-89 had fewer new sites of pain than men undergoing EBRT or HBI	Leukopenia grade-3: 3.1 % in Sr-89 vs. 0 % EBRT; thrombocytopenia grade-3/4: 6.9 % in Sr-89 vs. 3.4 % in EBRT	Quilty et al. (1994)
200 MBq Sr-89 (76) vs. local EBRT (72).				
200 MBq Sr-89 (77) vs. HBI (80)				
Patients with metastatic HRPC	Subjective response using pain score, analgesic use or performance status	No differences in subjective pain responses, analgesic consumption, or performance status. Interestingly, overall survival rate of patients that received local EBRT was longer than those receiving Sr-89	No grade-3/4 leukopenia; one patient in Sr-89 with grade III toxicity	Oosterhof et al. (2003)
150 MBq Sr-89 (101) vs. local field EBRT (102)				
Patients with metastatic bone pain	Pain relief	Significant pain relief produced with Sr-89	Thrombocytopenia (grade 3 toxicity in 12 %, and grade 4 in 15.4 % of patients in Sr-89 treatment group	Lewington et al. (1991)
Sr-89 vs. placebo (26)				
Patients with metastatic prostate cancer	Pain relief	No significant difference in the analgesic effect between both radionuclides was found in the group of patients with prostate carcinoma	Moderate pancytopenia, granulocytopenia and/or thrombocytopenia were observed in both Sr-89 and Sm-153 group, with no significant between group differences	Baczyk et al. (2007)
150 MBq Sr-89 (30) vs. 37 MBq/kg Sm-153 (30)				
Patients with painful bone metastases	Pain relief	62–72 % of patients had pain relief with 1.0 mCi/kg during first 4 weeks and 31% had complete/marked relief by week 4	With 1.0 mCi/kg: grade-3/4 anaemia in 6 %, thrombocytopenia in 3 % and leukopenia in 14 % (compared to 35, 0 and 0 %, respectively, with placebo)	Serafini et al. (1998)
Sm-153 at 0.5 (40) or 1 mCi/kg (39) vs. placebo (39)				
Patients with metastatic HRPC	Pain relief	Sm-153 had positive effects on measures of pain relief compared with placebo within 1–2 weeks, and also reduced opioid consumption by week 3. There was no significant difference in survival	Grade 3 thrombocytopenia and leucopenia were noted in 3 and 5 % of patients, respectively, in the active treatment arm	Sartor et al. (2004)
1 mCi/kg Sm-153 (101) vs. placebo (51)				
Patients with painful bone metastases	Pain relief	At week 4 after dose administration, statistically significant pain relief was produced by 1.0 mCi/kg dose of Sm-153	Values for platelets and WBCs reached nadirs at 3 or 4 weeks with both doses and recovered by 8 weeks	Resche et al. (1997)
Sm-153 at 0.5 mCi/kg (55) vs. 1.0 mCi/kg (59)				
Patients with prostate cancer-induced bone pain	Number of positive pain response days	Mean percentage of pain response days 27 % (Re-186) vs. 13 % (placebo). The number of patients who requested radiotherapy was higher in the placebo group (67 %) than in the Re-186 group (44 %). Re-186 resulted in a significantly longer pain response in the treatment of bone pain from metastasized prostate cancer	Death of five patients in rhenium group due to clinical deterioration of patient’s condition	Han et al. (2002)
12 weeks treatment with 35–80 mCi Re-186 (59) vs. placebo (52)				
Patients with metastatic CRPC	Pain response	At week 8 there were 40, 63, 56 and 71 % pain responders in the 5, 25, 50 and 100 kBq/kg groups, respectively, and of responders, 6/20 (30 %), 8/19 (42 %), 8/18 (44 %) and 11/21 (52 %) reached complete (pain index 1) or marked pain response (pain index 2), respectively. Mean pain relief duration was 44 days in the 50 and 100 kBq/kg groups, and 28 and 35 days in the 5 and 25 kBq/kg groups, respectively	Anaemia (11 %) and haemoglobin decrease (15 %) in all dose groups, with no significant differences between them. For 2 weeks post-injection of higher Ra-223 doses, there was a reduction in platelet, white blood cell and neutrophil counts, which later returned back to baseline	Nilsson et al. (2012)
16 weeks treatment with 5 (26), 25 (25), 50 (25) or 100 (24) kBq/kg i.v. Ra-223				
Patients with metastatic CRPC	PSA levels, bone alkaline phosphatase levels and pain responses	The study met its primary end point with a confirmed ≥50 % PSA response in 0 % patients receiving 25 kBq/kg, 6 % receiving 50 kBq/kg, and 13 % receiving 80 kBq/kg at 24 weeks. A ≥50 % decrease in bone alkaline phosphatase levels was identified in 16, 67, and 66 patients in the 25-, 50-, and 80-kBq/kg dose groups, respectively. Reduced pain responses were reported by 29–75 % of patients with baseline pain, with a trend towards greater response in the 50-kBq/kg dose group	The most common treatment-related AEs (≥10 %) occurring up to week 24 across all dose groups were diarrhoea (21 %), nausea (16 %), and anaemia (14 %). No differences in the incidence of hematologic events were seen among dose groups. In total, 70 deaths were recorded to 24 months after the first Ra 223 injection: 26, 22, and 22 deaths occurred in the 25-, 50-, and 80-kBq/kg dose groups, respectively	Parker et al. (2013)
Three intravenous injections of Ra-223 (25 (41), 50 (39) or 80 (42) kBq/kg) at 6-week intervals over 24 weeks				
Patients with metastatic CRPC	Overall survival, time to initial ERBT or opioid use	Ra-223 significantly improved overall survival in patients with CRPC (14 months), c.f. placebo (11.2 months). Time to EBRT was significantly longer in the Ra-223 group vs placebo. Median time to initial opioid use was significantly longer in the Ra-223 group, with a risk reduction of 38 % compared to placebo. Fewer patients in the Ra-223 group (36 %) than in the placebo group (50 %) required opioid use for pain relief	Safety and tolerability of Ra-223 were highly favourable and showed a low incidence of myelosuppression (grades 3/4 neutropenia in 1.8 % and 0.8 %, and thrombocytopenia in 4 % and 2 % of the Ra-223 and placebo groups, respectively)	Parker et al. (2012), Nilsson et al. (2013)
Six injections of Ra-223 at 50 kBq/kg every 4 weeks (614) vs. placebo (317)				
Bisphosphonates				
Men with metastatic HRPC	Skeletal-related events, time to the first skeletal-related event, skeletal morbidity rate, pain and analgesic scores, disease progression, and safety	At 15 months, zoledronic acid at 4 mg significantly reduced the mean increase from baseline in pain score and skeletal-related events in patients with prostate induced bone metastases	Zoledronic acid at 4 mg given as a 15-min infusion was well tolerated, but the 8 mg dose was associated with renal function deterioration	Saad et al. (2002, 2004)
Intravenous zoledronic acid at 4 mg (214) or 8 mg (221) vs placebo (208) every 3–4 weeks				
Patients with bone metastases	Pain relief	Significantly reduced mean VAS pain score from baseline. Zoledronic acid 4 mg administered as a 15-min infusion every 3–4 weeks was well tolerated, including patients who had significant prior exposure to bisphosphonate	Fatigue, nausea, and arthralgia	Vogel et al. (2004)
Intravenous zoledronic acid at 4 mg (638) every 3–4 weeks for six doses				
Men with metastatic bone pain	Pain relief	There were no sustained significant differences between the pamidronate and placebo groups in self-reported pain measurements or analgesic use at either week 9 or 27	Overall, pamidronate disodium was well tolerated	Small et al. (2003)
Intravenous pamidronate at 90 mg (180) or placebo (194) every 3 weeks for 27 weeks				

Adapted from Goyal and Antonarakis (2012), Lipton (2007)

*Sr-89* strontium-89, *Sm-153* samarium-153, *Re-186* rhenium-186, *Ra-223* radium-223, *QoL* quality of life, *QLQ C30* Quality of Life Questionnaire of the European Organization for Research and Treatment of Cancer, *WHO* World Health Organization, *HBI* hemi-body irradiation, *EBRT* external beam radiotherapy, *HRPC* hormone-refractory prostate cancer, *CRPC* castration-resistant prostate cancer, *AEs* adverse effects, *PSA* prostate-specific antigen, *RTOG* Radiation Therapy Oncology Group, *VAS* Visual Analogue Scale

### Metastasis or tumourigenesis inhibitors

In the next section, we provide an overview of therapeutic agents shown to slow the progression of prostate cancer-induced metastasis to the skeleton; detailed reviews may be found elsewhere (see Russo et al. 2010; Saad and Lipton 2010b; Saylor et al. 2013). As the analgesic efficacy for most of these agents in patients with PCIBP has not been reported on, this is a knowledge gap that remains to be addressed.

#### RANKL inhibition

RANK/RANKL/OPG signalling plays a key role in the early stages of PCIBP (Castellano et al. 2011). OPG is suggested to be a promising agent for the treatment of PCIBP that acts by reducing osteoclast function to diminish tumour-induced bone destruction (Body et al. 2003). However, OPG is poorly selective and also inhibits TRAIL, which promotes tumour cell apoptosis (Neville-Webbe et al. 2004).

Denosumab (AMG 162) is a human monoclonal IgG_2_ antibody directed against RANKL, with an extremely high affinity for human RANKL (Schwarz and Ritchlin 2007). With a greater decrease in bone marker turnover and a longer duration of action, AMG 162 is more potent than AMGN-0007, a recombinant OPG. A large Phase III randomized clinical trial demonstrated the superiority of denosumab over zoledronic acid in prevention of SREs in men with bone metastases and CRPC (Fizazi et al. 2011). Based on these findings, denosumab (Xgeva™, Amgen Inc.) was approved by the US FDA for the prevention of SREs in patients with bone metastases from solid tumours.

#### Endothelin-1 antagonists

The importance of the ET axis in cell growth, invasion, regulation of apoptosis, and stimulation of angiogenesis has led to the concept of ET antagonism (Lalich et al. 2007). The orally active ET_A_R antagonist, atrasentan (ABT-627, Xinlay™, Abbott) (Opgenorth et al. 1996), attenuated disease progression as well as reduced morbidity in patients with PCa-induced bone metastases (Lalich et al. 2007). Phase I clinical trials of atrasentan reduced pain in 70 % of patients when evaluated using a VAS (Visual Analogue Scale), and decreased PSA levels in ~45 % of patients (Carducci et al. 2002; Zonnenberg et al. 2003; Ryan et al. 2004).

Phase II clinical trials of atrasentan reduced PSA levels and significantly delayed disease progression (Carducci et al. 2003; Nelson et al. 2003). In addition, expression levels for markers of bone formation and resorption mirrored the preclinical data (Carducci et al. 2003; Nelson et al. 2003). Although subsequent Phase III clinical trials of atrasentan in men with metastatic hormone-refractory PCa (mHRPC) produced favourable trends for time to PSA progression and change in bone alkaline phosphatase levels, there was no delay in disease progression (Carducci et al. 2007; Nelson et al. 2008).

Zibotentan (ZD-4054, AstraZeneca) is an endothelin antagonist that reportedly has a beneficial impact on PCa progression and overall patient survival (James et al. 2009; Schelman et al. 2011). A preliminary clinical study of the safety and efficacy of ZD4054 (Zibotentan) in combination with docetaxel (Taxotere) in patients with metastatic HRPC showed a favourable safety and tolerability profile for this drug combination in patients with metastatic HRPC (Trump et al. 2011).

#### Src inhibition

Src, a non-receptor tyrosine kinase, is the prototypic member of the Src-family of kinases (SFKs) (Summy and Gallick 2003). SFKs are components of signal transduction pathways involved in normal cellular growth, proliferation, angiogenesis and motility, which when deregulated promote tumour progression (Kim et al. 2009b). Over-expression of Src in osteoclasts (Horne et al. 1992) has been linked to cancer progression (Asim et al. 2008). PTHrP and IL-8 are important mediators in bone metastases that activate the androgen receptor which is implicated in Src signalling (Lee et al. 2001, 2004; DaSilva et al. 2009). Given these roles for Src, it is not surprising that Src inhibitors have emerged as therapeutics in the treatment of PCa (Kim et al. 2009b). Several in vitro and in vivo studies have demonstrated potential anti-tumour and anti-osteoclast activity of the Src inhibitors, dasatinib (BMS-354825, Bristol-Myers-Squibb) and saracatinib (AZD-0530, AstraZeneca) (Nam et al. 2005; Chang et al. 2008; Park et al. 2008; Vandyke et al. 2009; Brownlow et al. 2009; Araujo et al. 2009; Koreckij et al. 2009). Dasatinib (Sprycel^®^, Bristol-Myers-Squibb) is approved for treatment of imatinib-resistant chronic myelogenous leukaemia and Philadelphia chromosome-positive acute lymphoblastic leukaemia (FDA 2010).

In a phase I clinical trial, dasatinib prevented apparent cancer progression in 43 % of patients and 51 % of patients achieved a ≥40 % reduction in levels of uNTX (Araujo et al. 2012). In a subsequent Phase II clinical trial, there was a ≥35 % reduction in levels of uNTX in 49 % of patients and a significant reduction in BAP and PSA levels in 73 and 59 % of patients, respectively (Yu et al. 2009, 2011). Two different dosage schedules of dasatinib were evaluated in a phase II clinical trial and a phase II extension trial in patients with metastatic CRPC who had not received prior chemotherapy (Yu et al. 2009, 2011). These studies showed a reduction in levels of uNTX and BAP (Yu et al. 2009, 2011). Phase I and II clinical trials of saracatinib also showed a positive correlation with respect to cancer progression and PSA levels (Lara et al. 2009; Hannon et al. 2010). The results of a Phase III clinical trial of dasatinib (NCT00744497) are awaited, which will provide the first opportunity to more fully assess the potential of Src inhibition as a strategy to extend survival in patients with PCa-induced bone pain.

KX2-391 (Kinex Pharmaceuticals) is a specific Src inhibitor (Naing et al. 2013) that binds to the peptide substrate-binding site of Src rather than its ATP-binding site (Naing et al. 2013). A recently completed single-arm Phase II clinical study that evaluated the efficacy of KX2-391 in men with bone metastatic CRPC who had not received prior chemotherapy (NCT01074138) showed a modest reduction in levels of bone turnover markers (Antonarakis et al. 2013). However, it failed to show anti-tumour activity at the dose (40 mg twice daily) of KX2-391 evaluated (Antonarakis et al. 2013).

### New therapeutics in development for alleviation of PCIBP

#### Cathepsin K inhibitor

Cathepsin K is a lysosomal cysteine protease secreted by osteoclasts that degrades the extracellular matrix during the process of bone resorption (Wilson et al. 2009). Its significance in bone remodelling is evident by the osteopetrotic phenotype observed in cathepsin K-null mice (Saftig et al. 1998). Expression of cathepsin K has been found in many malignancies, including prostate and breast cancers (Brubaker et al. 2003; Littlewood-Evans et al. 1997). The only cathepsin K inhibitor studied in humans, odanacatib (MK-822, Merck), is still in its early stages of development (Rachner et al. 2012). Although the efficacy of odanacatib is as yet unknown in patients with prostate cancer, odanacatib has shown promising results in clinical trials of osteoporosis in post-menopausal women (Stoch et al. 2009; Bone et al. 2010; Eisman et al. 2011) and in patients with breast cancer-induced metastatic bone disease (Jensen et al. 2010).

#### Integrin inhibitors

Integrins are heterodimeric adhesion receptors that regulate cell adhesion, migration, invasion and motility (Millard et al. 2011). The integrins, αvβ3 and αvβ5, are involved in metastases in men with PCa (Seftor et al. 1992; Knox et al. 1994; Schneider et al. 2011). The integrin, αvβ3, is the most abundant in osteoclasts and is critical in osteoclast formation and activity (Clover et al. 1992; Nakamura et al. 2007). Antibodies that bind and block αvβ3, inhibit bone resorption (Nakamura et al. 2007). Vitaxin^®^ (MEDI-522), a humanized monoclonal antibody that blocks αvβ3 integrin, is in early clinical development for metastatic melanoma and PCa (Gramoun et al. 2007). In a small multicenter, randomized, double-blind clinical study involving 21 patients with bone metastases and metastatic HRPC, an orally active non-peptide small molecule inhibitor of αvβ3, MK-0429, was generally well tolerated with evidence of an early reduction in bone turnover; a common side effect was nausea (Rosenthal et al. 2010). However, the short duration of treatment (4 weeks) made it difficult to draw conclusions with respect to drug efficacy (Rosenthal et al. 2010). Hence, clinical trials involving larger numbers of patients with efficacy assessed over a longer period are required to evaluate the potential clinical use of αvβ3 inhibitors in the treatment of metastatic bone disease and/or PCIBP.

#### Sclerostin

Sclerostin is a secreted cysteine-knot protein of the differential screening-selected gene aberrant in the neuroblastoma (DAN) family, which includes proteins that antagonize BMP and Wnt signalling (Moester et al. 2010). Although the mechanism by which sclerostin negatively regulates bone formation is still an enigma, sclerostin inhibits differentiation and function of osteoblasts by binding to the first β-propeller of the low-density lipoprotein-related protein (LRP5/6) (Lin et al. 2009). Sclerostin knockout mice have greater bone mass and bone strength due to increased bone formation (Li et al. 2008). A sclerostin antibody (AMG-075, Amgen) is currently in clinical trials (Marenzana et al. 2011; Lewiecki 2011; Tian et al. 2010; Agholme et al. 2010; Eddleston et al. 2009; Li et al. 2009) and so may also have potential as a novel therapeutic for alleviation of PCIBP.

A significant limitation of the aforementioned clinical trials of new treatments for slowing the progression of prostate cancer-induced skeletal metastases is between-study variability in clinical trial endpoints with pain assessments rarely included. Standardization of clinical trial endpoints would facilitate between-treatment comparisons and the development of evidence-based treatment guidelines. With regard to palliative radiotherapy, the International Bone Metastases Consensus Working Party has addressed this issue with recent publication of a set of standard clinical trial endpoints that include assessments of pain and quality of life (Lemke et al. 2012). Widespread adoption of standardized clinical trial endpoints that include assessment of pain and treatment impact on quality of life measures for evaluation of new therapies aimed at slowing the progression of skeletal metastases would greatly facilitate the identification of those treatments that improve patient outcomes.

## Conclusion

The pathobiology of prostate cancer-induced bone pain is complex involving components of neuropathic, inflammatory and ischemic pain arising from ectopic sprouting and sensitization of primary afferent sensory nerve fibres within prostate cancer-invaded bones. Dynamic cross talk between metastatic cancer cells, cellular components of the bone matrix (osteoblasts and osteoclasts) and factors associated with the bone microenvironment contribute to the establishment and maintenance of PCIBP. Hence, it is not surprising that the clinical management of PCIBP requires multimodal treatment involving radiotherapy, analgesics (opioids, NSAIDs), bisphosphonates, radioisotopes and tumouricidal therapies. Further research to gain a deeper understanding of the molecular mechanisms underpinning the bidirectional cross talk between the various elements contributing to the pathobiology of PCIBP is required. The knowledge so gained will be invaluable in guiding drug discovery programs aimed at producing a new generation of efficacious and well-tolerated analgesic/adjuvant agents for improved relief of intractable pain in patients with advanced skeletal metastases.
